# Standardization developments for large scale biobanks in smoking related diseases - a model system for blood sample processing and storage

**DOI:** 10.1186/2213-0802-1-14

**Published:** 2013-08-30

**Authors:** Johan Malm, Thomas E Fehniger, Pia Danmyr, Ákos Végvári, Charlotte Welinder, Henrik Lindberg, Paul Upton, Stephanie Carter, Roger Appelqvist, Karin Sjödin, Elisabet Wieslander, Magnus Dahlbäck, Melinda Rezeli, David Erlinge, György Marko-Varga

**Affiliations:** 1 Department of Laboratory Medicine, Section for Clinical Chemistry, Lund University, Skåne University Hospital in Malmö, Malmö, 205 02 Sweden; 2 Clinical Protein Science & Imaging, Biomedical Center, Dept. of Measurement Technology and Industrial Electrical Engineering, Lund University, BMC C13, 221 84 Lund, Sweden; 3 Region Skåne R&D Center, Region Skåne, 221 85 Lund, Sweden; 4 Department of Oncology, Clinical Sciences, Lund University, 221 85 Lund, Sweden; 5 Region Skåne Biobank, Skåne University Hospital, 221 85 Lund, Sweden; 6 Thermo Fisher Scientific, Stafford House, 1 Boundary Park, Hemel Hempstead, Hertfordshire HP2 7GE UK; 7 Thermo Fisher Scientific, Rochester, MA 14625 USA; 8 Respiratory and Inflammation Therapy Area, Astra Zeneca R&D, 431 83 Mölndal, Sweden; 9 Department of Cardiology, Lund University, Skåne University Hospital, 221 85 Lund, Sweden; 10 First Department of Surgery, Tokyo Medical University, 6-7-1 Nishishinjiku Shinjiku-ku, Tokyo, 160-0023 Japan

**Keywords:** Biobank, Biomarkers, Standardization, Robotics, Respiratory diseases

## Abstract

**Background:**

Biobank samples stored in biobanks give researchers and respiratory healthcare institutions access to datasets of analytes valuable for both diagnostic and research practices. The usefulness of these samples in clinical decision-making is highly dependent on their quality and integrity. New procedures that better preserve sample integrity and reduce degradation are being developed to meet the needs of both present and future biobanking. Hereby we present an automatic sample workflow scheme that is designed to handle high numbers of blood samples.

**Methods:**

Blood fractions are aliquoted, heat sealed using novel technology, and stored in 384 tube high-density sample arrays.

**Results:**

The newly developed 384 biobank rack system is especially suited for preserving identical small aliquots. We provide data on robotic processing of clinical samples at −80°C, following initial processing, analysis and shipping between laboratories throughout Europe. Subsequent to unpacking, re-sorting, and storage at these sites, the samples have been returned for analysis. Biomarker analysis of 13 common tests in the clinical chemistry unit of the hospital provides evidence of qualitative and stable logistics using the 384-sample tube system.

**Conclusions:**

This technology development allows rapid access to a given sample in the frozen archive while maintaining individual sample integrity with sample tube confinement and quality management.

**Electronic supplementary material:**

The online version of this article (doi:10.1186/2213-0802-1-14) contains supplementary material, which is available to authorized users.

## Background

The future of biomedical sciences will be driven by the ability to adopt novel technologies, generating large data sets of data, to understand disease and to develop new treatments. This is especially relevant to diseases such as lung cancer (LC) and chronic obstructive pulmonary diseases (COPD), responsible for high mortality and cost to the European health care system. Cancer, and especially lung cancer, as well as chronic obstructive pulmonary disease (COPD) are leading causes of smoking related mortality worldwide. Overall, a therapeutic plateau has been reached with conventional anti-cancer treatment strategies such as cytotoxic agents, radiotherapy and surgery. Given the still dismal survival rates, attention in recent years has focused on novel molecular targeted therapies with different mechanisms of action and better toxicity profiles. Accordingly, there is a clinical need to: (i) identify biomarkers that can help recognize patients that respond to these therapies, (ii) detect tumor resistance, (iii) and predict the efficacy of targeted drugs cost-effectively. COPD is a progressive debilitating disease that often is not diagnosed before extensive tissue destruction has occurred. Prevalence rates of COPD vary across EU, ranging from 4 to 10% with an overall rate of respiratory symptoms between 45-65% across EU. Currently COPD is under-diagnosed and is associated with co-morbidity and risk for that increase the burden on the healthcare system, hospitalization costs and mortality. By implementing early detection, personalized treatment and evaluation of response to treatment both Lung Cancer and COPD have led to improved prognosis and reduced cost to the healthcare system. These smoking related respiratory diseases are multifactorial. Meaning that not a single molecule is related to one disease or clinical complaint, but rather to hundreds of molecules that are interconnected or pathway related in addition to protein complex formed. There is therefore a need for selecting from multiple read-out signals. However, this is hampered by the lack of tools and data for early diagnosis. One new direction that is proving to be a winning concept is the modeling of disease progression and evaluation of treatment responses.

Biobanking is gaining momentum with an increasing number of valuable patient samples linked with clinical information that are being stored in biobanks around the word [1, 2]. The advancement in clinical research where high quality samples are utilized in research has increased significantly in the recent decade, but especially in the last years [3–10]. The targeted treatments with Personalized Medicines are becoming the new generation of drugs with high specificity and safety. In the developments of these new drugs, high quality patient samples are mandatory, where standardized Biobank resources will play a major role [4, 11]. It has resulted in interest to share data, facilitated by biorepositories of specimens that is linked to health care information, where a lot of effort is given to meet the governance challenges of Biobank archives now and in the future [12] *.* Sample integrity is a real challenge, but also a mandatory requirement for any patient read out. In addition, life science clinical research material needs to use standard procedures, optimized protocols and processing cycle times, which are key aspects of work flows to consider [13, 14]. Protein and molecular degradation is a well-known phenomenon that is directly related to sample handling procedures, where sample temperature, preparation times and sample preparation procedures are some variables that will be directly related to sample stability [15, 16]. This is especially important when samples are stored frozen in 5–12 ml tubes, thawed and collected for analysis and then re-frozen again for future use. When this procedure is repeated multiple times, the composition of the original sample state is irreversibly changed. This gives rise to changes in absolute amounts of analytes that is not related to, *e.g.*, the disease state of the patient sample. Processing times of blood might vary in-between some hours to 24 and 36 hours. The read-outs from samples that have been exposed to these extended processing times will vary. This is particularly critical to RNA analysis, metabolomics quantitation as well as protein expression analysis.

The processing protocols were worked out and reported in large-scale studies, such as the UK Biobank (http://www.ukbiobank.ac.uk/) and LifeGene (https://www.lifegene.se/). Keeping an absolute control of patient sampling, standardized procedures, validated sample handling protocols and electronic surveillance of the sample life cycle ranging from the needle in the arm of the patient to the automated storage handling at −80°C will provide the basis for high quality biobanks [2, 14, 17]. New technology is changing a lot of the way that standard procedure in hospitals is performed. Sample degradation and sample losses due to evaporation along with cot effective sample archiving are among the long-standing issues that are most important in order to keep high quality of samples for long periods.

By the implementation of e-health logistics, efficient data storage and use, allows a data history to be established where the patient treatments are linked to the decision making of the physician, providing healthcare improvements [18]. A novel technology that relate to biomarkers and clinical status of the patients, is a great resource that utilizes blood samples as a major biofluid resource. Blood sample storages nowadays range from large national efforts into smaller development labs where the biospecimen collections are used as biobank assets, searching for healthcare solutions [19, 20].

The value that large scale biobanks provide paves the way for new areas that opens up the opportunities such as drug discovery, stem cell research and genetic research [21–23]. This study presents novel developments that improve the existing biobanking systems.

## Methods

### Materials and instruments

Matrix storage tubes and seals, 0.1 mL 384 2D tubes (Thermo Scientific 384 well 2D coded storage tubes part 3815, MA, USA). In the sealing experiments we used a WellMate dispenser (Thermo Scientific, MA, USA), a small bore tubing cartridge (Thermo Scientific, MA, USA). A 384 rack heat sealer (ALPS3000 Thermo Scientific, MA, USA), the Heat 20 μm sealing foil (Easypierce, Thermo Scientific, MA, USA), the PTFE Type K couples TM Electronics (RS 409–4908, Thermo Scientific, MA, USA), 8 channel USB data logger, and Picotech picolog software (Pico technologies USBTC08). A Hamilton STAR Liquid Handling Platform (Hamilton, Reno, NV) was employed for automated aliquoting of blood samples. CO-RE 480 standard volume tips (300 μL) with filters and CO-RE 480 standard volume tips (1000 μL) without filters were purchased from Hamilton (Bonaduz, Switzerland). The variable temperature heat sealer instrument (ALPS™ 50 V, Thermo Scientific, MA) was used for sealing 384-tubes with Easy Pierce 20 μm heat-sealing tape (AB-1720, Lot No: 115895) (Thermo Fisher Scientific, MA). For registration of aliquots a VisionMate® scanner was used (Thermo Fisher Scientific, MA). Nautilus LIMS (Thermo Fisher Scientific, MA) was used throughout the entire study.

### Blood samples

Blood samples were provided by healthy volunteers at the Skåne University Hospital, Malmö, Sweden. Samples were collected in the morning after breakfast intake. 10 mL blood was sampled repeatedly in primary tubes. EDTA sample types were centrifuged for 10 minutes at 10,000 rpm, which is the standard procedure at the hospital. 70-μL aliquots were dispensed into 384-rack tubes (volume of 100 μL). These samples tubes were stored at −80°C throughout the entire study, and on dry ice upon transportation.

The collection of blood samples was approved by the ethical board at Lund University (approval number: LU 532–03).

### Biomarker analysis

All analyses were performed by experienced clinical chemistry staff at the Department of Clinical Chemistry, Skåne University Hospital, Malmö, Sweden. The laboratory is accredited by SWEDAC (Swedish Board for Accreditation and Conformity Assessment). The following tests were run using standard methods from Roche: α_1_-antitrypsin, ALAT (alanine aminotransferase), albumin, apoA (apolipoprotein A1), creatinine, CRP, cystatin C, estradiol, Fe, ferritin, bile acid, glucose, fibrinogen, haptoglobin, HCG, IgA, IgG, IgM, K, Mg, Na, EPK, LPK, TPK, TSH, total protein. All samples were analyzed on a Cobas 8000 modular analyzer from Roche (Basel, Switzerland).

## Results and discussion

As the sample volume requirements are steadily decreasing within the clinical chemistry departments in hospitals around the world, blood sample handling from patients becomes a critical part in the healthcare logistics. We recently presented a 384 high density solution [17] that is currently being implemented as an efficient and cheap solution to Biobank archiving in clinical hospitals. The 384-rack is confined with a hard polymeric frame where the tubes (384) are mounted. Manual or robotic picking and sorting can be made for all of the tubes within the 384 biobank sample plate (10). The plates and foils have been pre-tested for the used conditions to make sure that there is no release of, *e.g.*, plastifiers to contaminate the samples.

**Figure 1:**
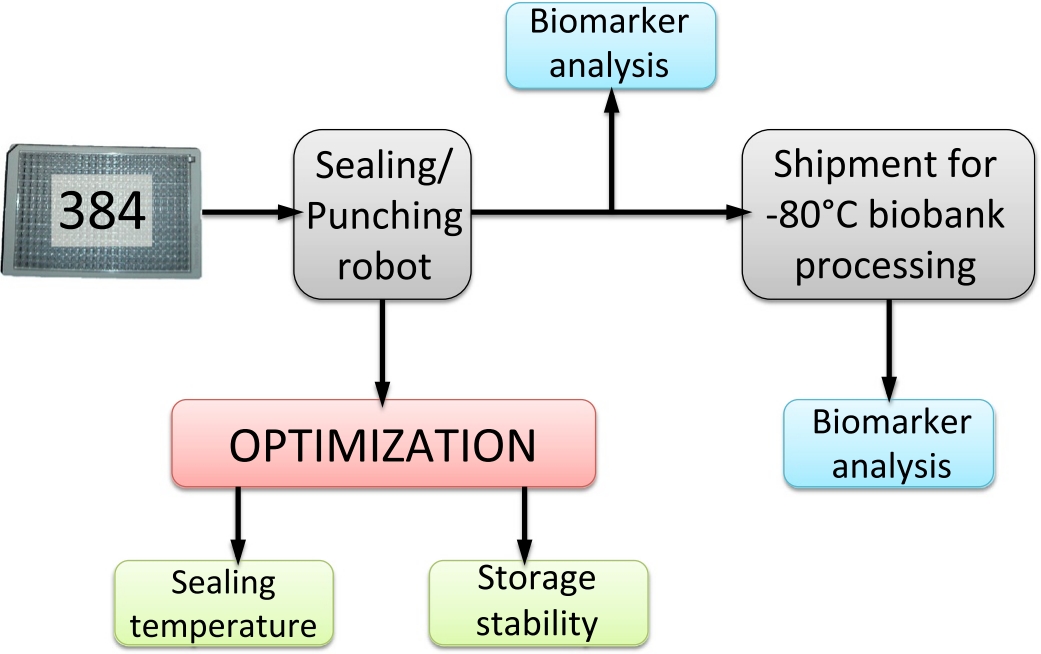
**Schematic illustration of the blood sample stability procedure conducted in order to investigate biomarker stability.**

Here we provide standardization and stability data on the development of 384 Biobank sample systems, including an entire cycle of blood plasma samples that were processed and sealed in the hospital in Malmö, Sweden. The samples were stored at −80°C, transported throughout Europe to Lichtenstein, processed by −80°C robotics and shipped back to the hospital in Malmö, as outlined in Figure 1.

### Sample tube confinement and quality management

In order to be efficient and reliable, biobanks must adopt and implement best practices with acceptable and standardized sample tube processing for Biobank storage.

A 384 Sealer-Cutter (Thermo Fisher Scientific) was evaluated, providing experimental evidence on sample temperature maintenance throughout the 384-rack sealing process utilizing sealing temperatures >150°C. One important question in these optimizations was: Are foil seal tubes stable at low temperatures?

We ran the experiments at room temp, and storage after liquid nitrogen storage over a period of 30 days, and with a sample number of n?=?30 or 16 for each measured points. By positioning probes in the tubes, the probes were placed in the tube just below the meniscus of the liquid. The probes were inserted from the side to reduce the displacement of sample in the well. The probe wire was routed out of the rack down an adjacent tube. The base and one side of the tube are removed to allow the wire to pass through. The rest of the tube is kept where possible in tact to keep the thermal mass of the test rack as close to a standard unmodified rack as possible. Probe wires must be routed between tubes or around the skirt of the rack to allow the rack to sit on the base of the tubes during the sealing process. Holes in one end of the rack skirt allow the wires to exit the side of the rack without interfering with the sealing process.

**Figure 2:**
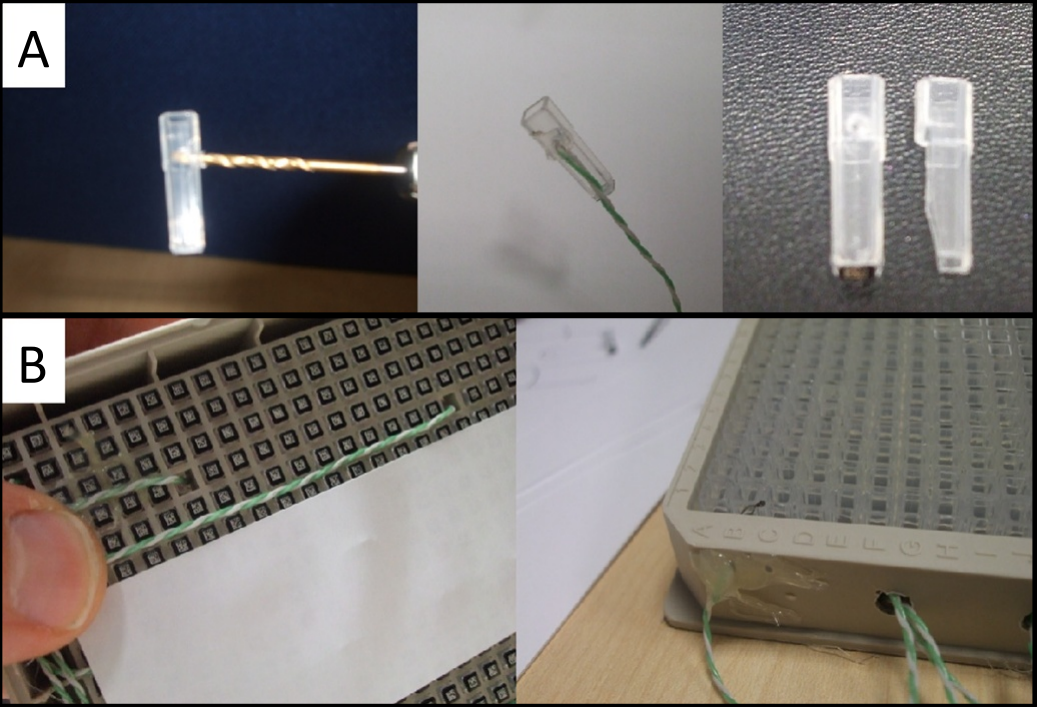
**Photos showing the tube preparation procedures for the temperature stability experiments (A) and photos showing the mounting of the temperature control of the 384-tube system (B).**

We performed a tube preparation i) by removing two adjacent tubes from the rack; then ii) drilling a 1-mm hole in one just above the ridge in the side. Next we removed the side and base of the other tube to the same height leaving the top of the tube intact. The probe wiring will exit through the cut away tube as shown in upper and lower images of Figure 2.

In order to investigate the temperature effect on the liquid within the 384 tube sets, the datalogger was set to log single measurements from the eight probes at 200 ms intervals and the log started prior to filling the racks. Next, tube racks were filled with 70 μL of water. For the wells containing temperature probes the liquid may bridge across the well above the probe. It is necessary to use a disposable tip to break the surface tension below the probe so the liquid drops to the bottom of the tube. The filled rack was then transferred to the ALPS 3000 and heat sealed, while sealing the door of the instrument was held open to allow the wires to prevent the wires getting trapped. Two test runs were made sealing at 165°C for 1 second and then for 2 seconds. The same rack was used with the foil removed for the second run. Further testing suggests sealing at 155°C for 0.5 seconds provides adequate sealing without deforming the tubes unduly. Sealing at 165 for 1 second melted more of the tube than is desirable. Fine-tuning and adjustments can be made at site and optimized accordingly.

**Figure 3:**
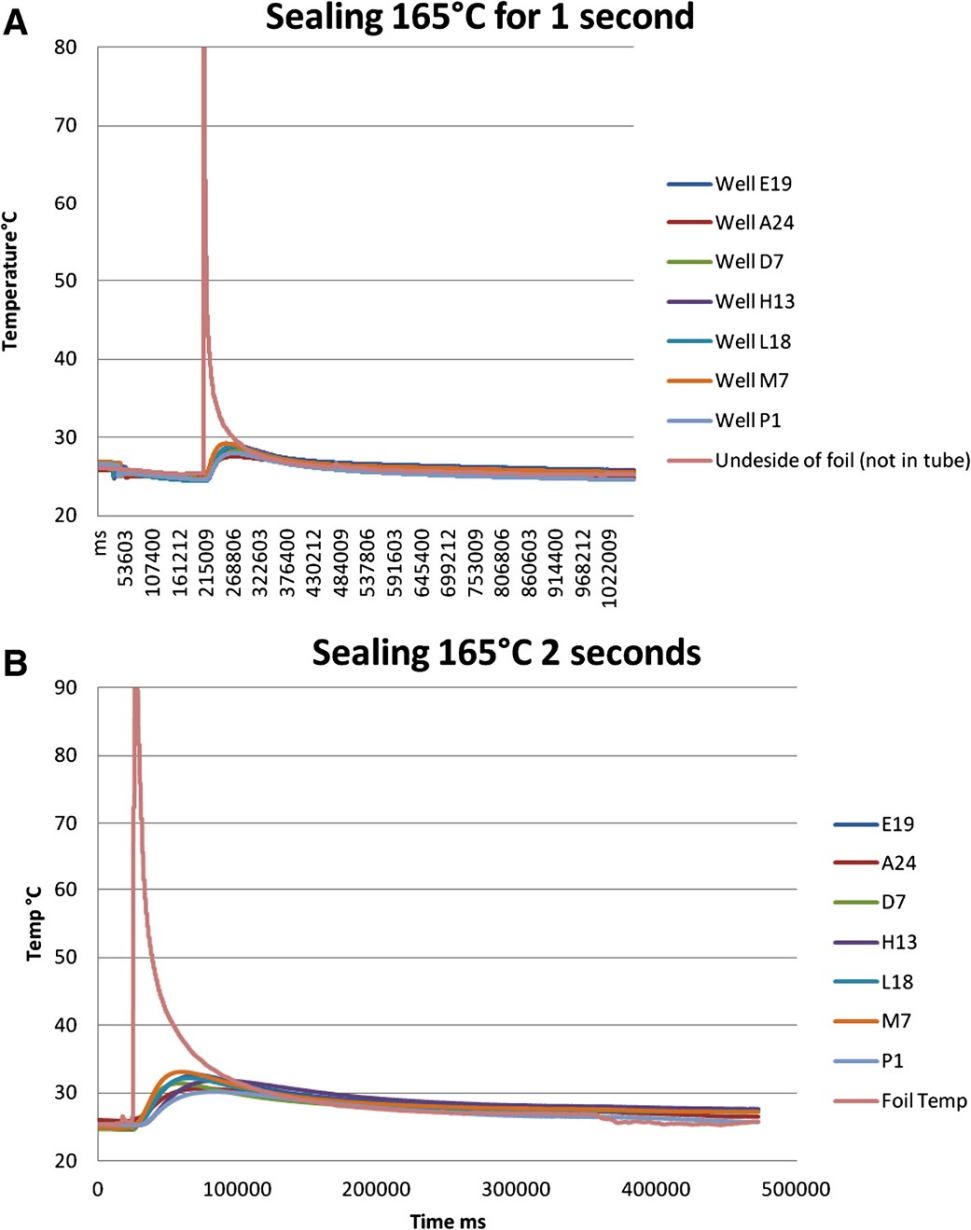
**Temperature dependence of automated sealing of the 384 sample tube systems at 165°C for (A) 1 and (B) 2s.** The different colors correspond to wells located in different regions of the rack.

The sample heating effects were investigated and presented in Figure 3A and 3B with 1 and 2 seconds of contact time at 165°C. The heat transfer effects will be minimal with above conditions. Time will be sufficient for the heat to diffuse from the foil to the sample. This result in that the system is efficiently cooled before much of the heat is transfers to the sample.

Figure 3 shows the temperature profiles upon sealing. It can be seen from Figure 3B, that the temp rises to about 32°C, and by lowering the time to 1 second it reaches about 28°C. This small elevation of temperature will quickly return to ambient temperature as shown in Figure 3.

The overall temperature dependencies are shown in Table 1 and these results indicate that the recommended sealing conditions for ALPS3000, 155°C for 0.5 seconds.

**Table 1:** **Sealing temperature dependence**

Sealing condition	Maximum average increase	Time to max average	Absolute max increase in individual well	Time to max in individual well
165°C/1 second	3.79°C	59 s	4.53°C	45 s
165°C/2 second	6.47°C	52 s	8.39°C	34 s

### Long term blood sample stability

Long term stability of samples with respect to samples volumes and evaporation in any storage temperature is of major importance. Simply because a decreased volume and alteration in sample volumes in Biobank tubes will result in false diagnosis quantitations.

**Figure 4:**
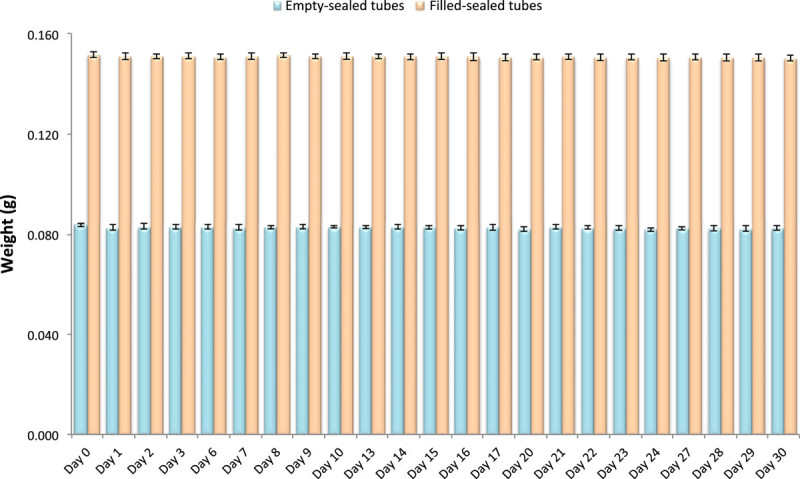
**Stability of filled (n = 30) and empty (n = 16) sealed sample tubes over 30 days.**

In order to investigate the tightness of our newly developed sealing, and the automated processing of 384 systems, we compared the weight of empty tubes, sealed empty tubes and tubes sealed with water inside. By comparing the data at ambient temperatures the stability was investigated over a 30 day time period. We found in the case of the sealed tubes both empty or filled the results were found to be highly similar, providing evidence that the evaporation from the 384 high-density biobank tube system insulates well after the sealing process (Figure 4). When calculating accelerated time frames in order to be able to extrapolate long-term stability, we have utilized the medical packaging accelerated ageing calculators (http://lso-inc.com/medical-package-testing/accelerated-aging.html). By using this calculation with the lower value (Q10), suggests that 1 day at 20°C is approximately equivalent to 1 year at −80°C.

### Robotic sample processing

The automated handling of samples in the biobank freezers are in most cases separated from the actual archiving of the sample racks. Robotic sorting and picking of sample tubes from the sample rack by most instruments are performed at −20°C. However, newly introduced robotic developments have proven to manage automated operations at −80°C. This is of great value, since the both the storage and the sample tube handling is performed at the same temperature (−80°C), where the samples are more stable.

Another sample stability consideration is the general principle that biobank samples are never re-used, *i.e.,* that the samples from biobanks are never re-entered back to −80°C storage. The reason for this strategy is to maintain the sample quality over time, which is impossible if the sample goes through repetitive freeze-thaw cycles. By utilizing high density 384-tube formats, with 70 μL sample volumes, the single use approach can be fulfilled [17]. In order to maintain the sample integrity and quality over time, the principle of single usage is also gaining acceptance.

### Ultra low temperature robotic and 384-tube processing

The sample integrity in clinical samples has to be maintained over longer time periods and provide a quality that can be used in global studies. The 384 high-density tube format provides this opportunity, and in order to be able to show the −80°C robotic processing procedures a pilot experiment was conducted. Plasma samples (n?=?768) in Sweden with racks of 384 tubes with a sample volume of 70 μL were shipped to Lichtenstein on dry ice. 13 biomarkers were quantified within the plasma samples in Sweden as a baseline. The same samples shipped to Lichtenstein, processed by the −80°C robotic picker and sorter (Kiwi, Liconic, Lichtenstein) in 10 cycles with a random sorting procedure. The robotic picker was operating constantly sorting the tubes in a random walk procedure within the 384-rack, from one position to the next. At no instance did the robotic arm drop or misplace the plasma sample tubes.

**Figure 5:**
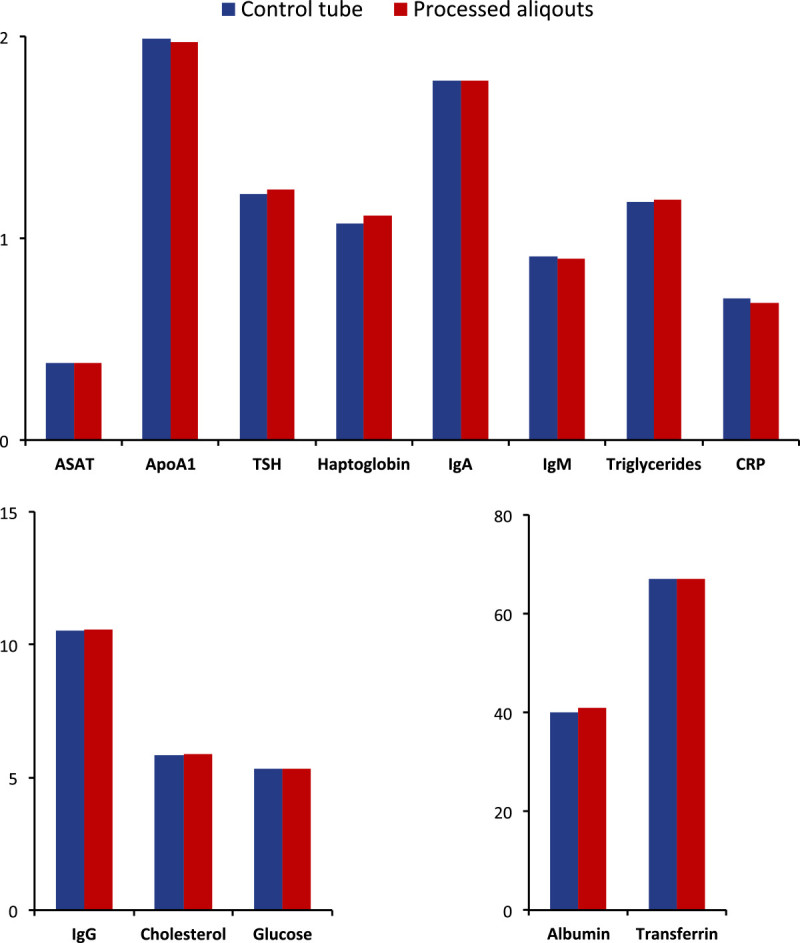
**Histograms of 13 biomarkers processed over a 7-week period using the 384-samples tube system.**

Next, the 384 plasma tube samples (n?=?45) were shipped back to the University hospital in Malmö on dry ice and analyzed using the 13 biomarker assays. These assays were chosen by the standards of highest frequency tests in the hospitals in southern Sweden where the patients with various diseases are screened. As shown in Figure 5, the difference in-between the two analysis are negligible, which points to the fact that the −80°C robotic processing does not influence the stability of blood plasma samples.

## Conclusions

The current manuscript is of major relevance to the diagnostic respiratory field as it outlines the standardization and qualitative aspects of blood sample processing in large-scale biobanking.

Biobanks are dependent upon effective sample processing and stable long-term low temperature storage systems. New methods for providing conditions of stable storage are currently being developed. Automatic robotic systems will be an essential component for the success of standardization procedures that speak to the preservation of sample integrity. This will be especially needed in studies of large number patient cohorts where samples are collected and processed in multitudes of laboratories at national and global levels of study. High cost that is associated with investments in robotic instrumentation is a challenge in a high quality driven healthcare society. Large scale processing on the other hand puts extremely high demands on manual sample handling and is close to impossible. As ultra-low temperatures demands for increased power consumptions, the lower temperatures also brings another challenge along that might be a disadvantage and that is huge temperature alterations upon power cuts or other electronic problems that ultimately will change the temperature of the sample.

In addition to their usefulness in clinical decision making, stable biobanked samples are also of importance to drug development, biomarker identification, sentinel studies of health and disease in society in both academic and regulatory settings [24].

## Authors’ original submitted files for images

Below are the links to the authors’ original submitted files for images. Authors’ original file for figure 1
Authors’ original file for figure 2
Authors’ original file for figure 3
Authors’ original file for figure 4
Authors’ original file for figure 5
