# Supplementary figures and images for: Standardization developments for large scale biobanks in smoking related diseases - a model system for blood sample processing and storage
Source: Transl Respir Med. 2013 Aug 30;1:14. doi: 10.1186/2213-0802-1-14 (PMC6733428; doi:10.1186/2213-0802-1-14)

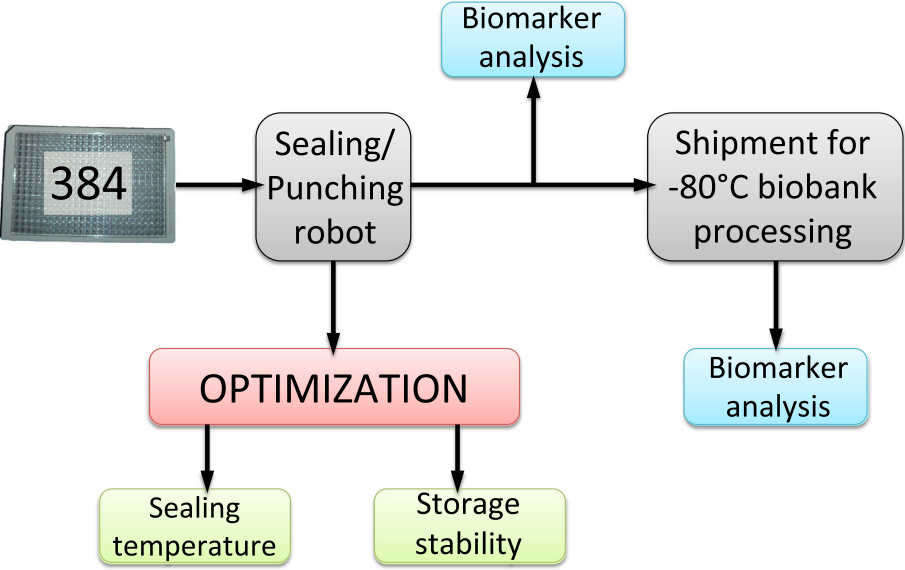

Supplement: Supplementary file 1 — Authors’ original file for figure 1 [file 40247_2013_14_MOESM1_ESM.pdf]

A

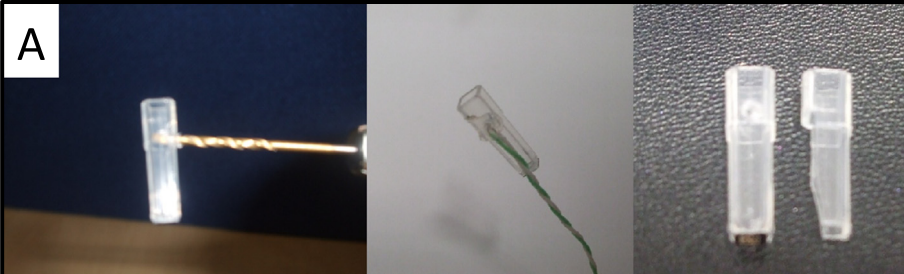

B

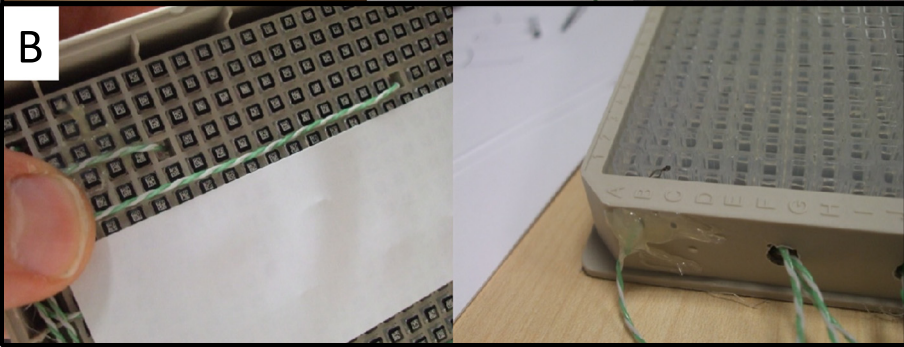

Supplement: Supplementary file 2 — Authors’ original file for figure 2 [file 40247_2013_14_MOESM2_ESM.pdf]

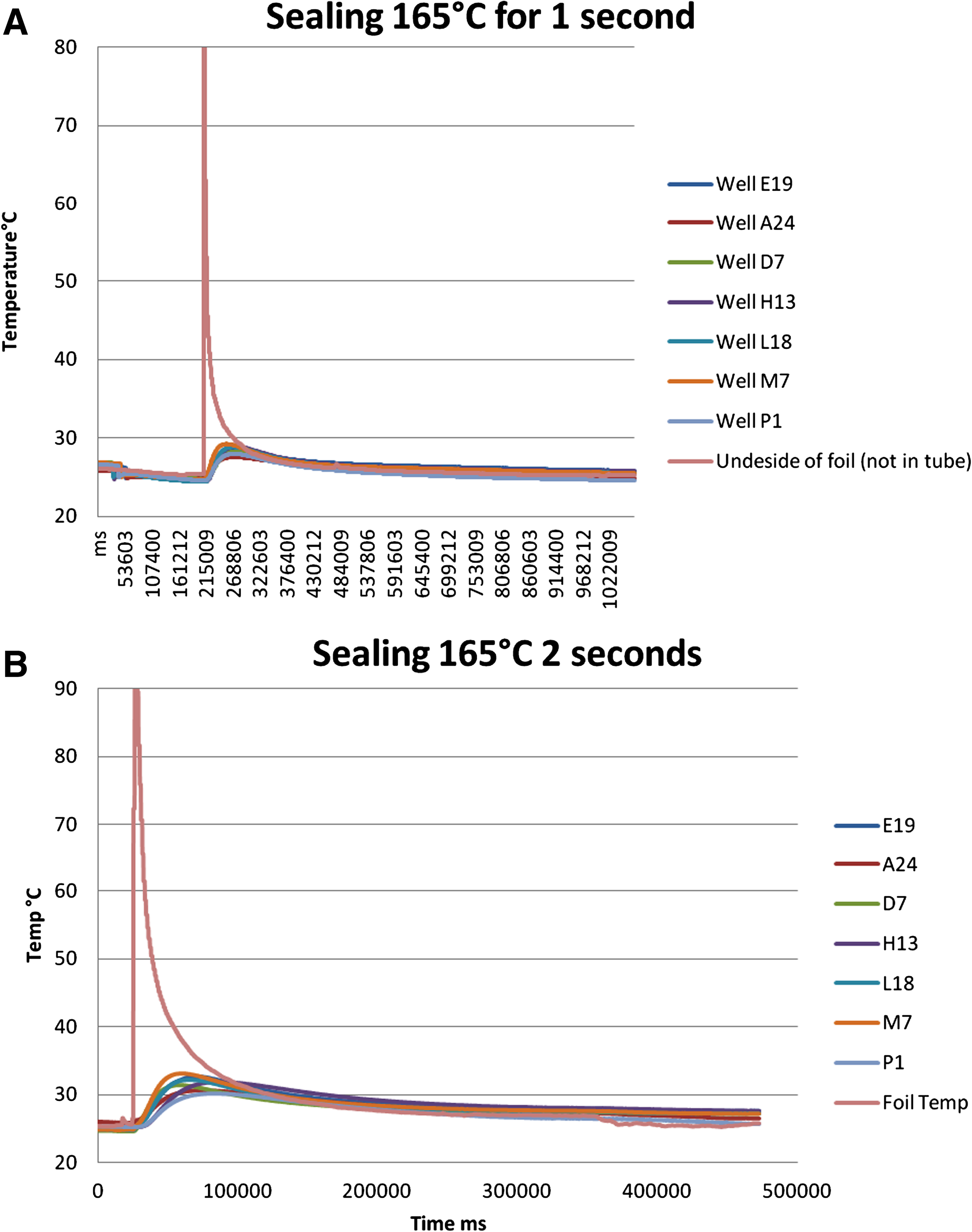

Supplement: Supplementary file 3 — Authors’ original file for figure 3 [file 40247_2013_14_MOESM3_ESM.tif]

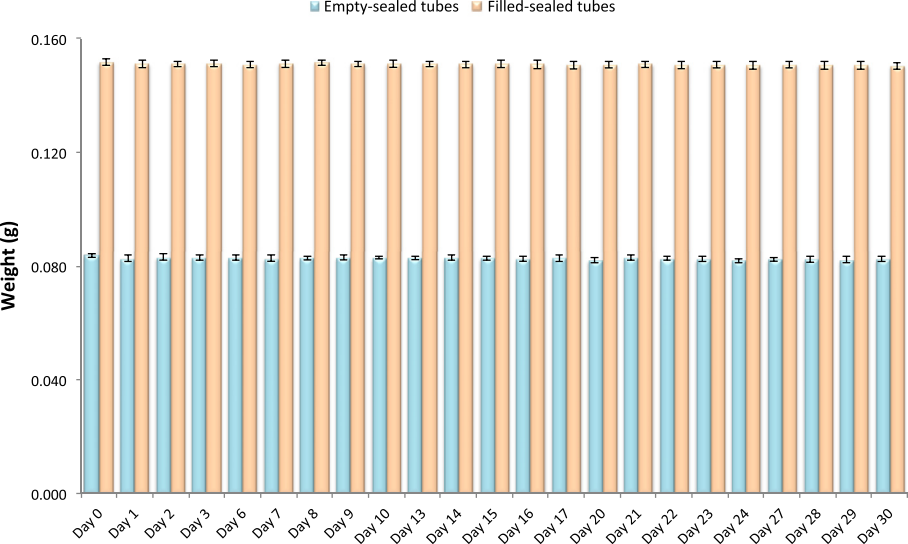

Supplement: Supplementary file 4 — Authors’ original file for figure 4 [file 40247_2013_14_MOESM4_ESM.pdf]

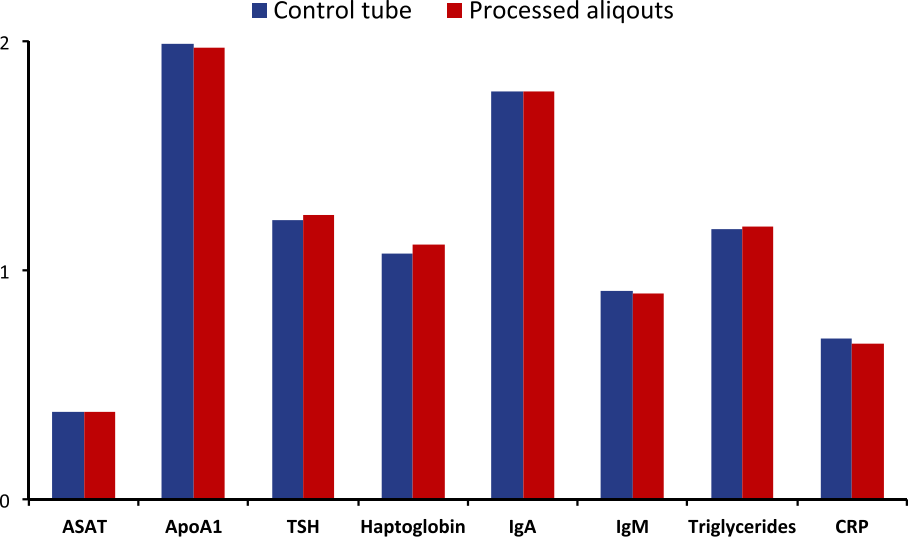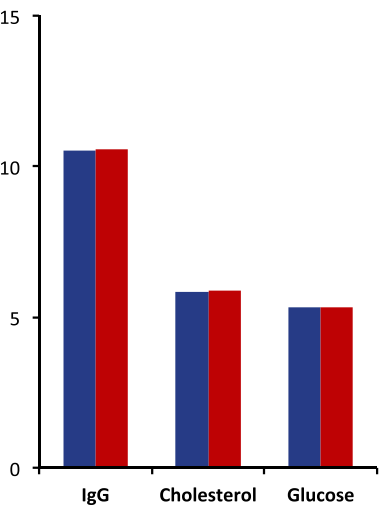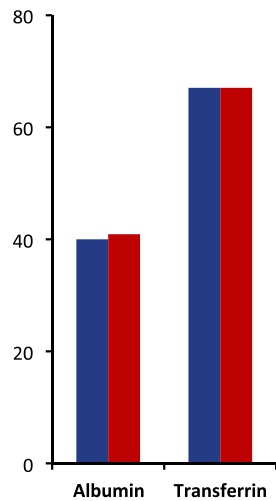

Supplement: Supplementary file 5 — Authors’ original file for figure 5 [file 40247_2013_14_MOESM5_ESM.pdf]
